# Kinematic and Diffusion Tensor Imaging Definition of Familial Marcus Gunn Jaw-Winking Synkinesis

**DOI:** 10.1371/journal.pone.0051749

**Published:** 2012-12-17

**Authors:** Antonella Conte, Francesco Brancati, Francesco Garaci, Nicola Toschi, Matteo Bologna, Giovanni Fabbrini, Marika Falla, Bruno Dallapiccola, Patrizio Bollero, Roberto Floris, Alfredo Berardelli

**Affiliations:** 1 IRCCS Neuromed, Pozzilli, Isernia, Italy; 2 Gabriele d’Annunzio University of Chieti-Pescara, Chieti, Italy and IRCCS Casa Sollievo della Sofferenza Hospital, Mendel Institute, Rome, Italy; 3 Department of Diagnostic Imaging, Molecular Imaging Interventional Radiology and Radiotherapy, “Tor Vergata” University of Rome, Rome, Italy; 4 IRCCS San Raffaele, Rome, Italy; 5 Medical Physics Section, Faculty of Medicine, “Tor Vergata” University of Rome, Rome, Italy; 6 Department of Neurology and Psychiatry, “Sapienza” University of Rome, Rome, Italy; 7 IRCCS Bambino Gesù Hospital, Rome, Italy; 8 Oral Pathology Unit, “Tor Vergata” University of Rome, Rome, Italy; University Hospital La Paz, Spain

## Abstract

**Background:**

Marcus Gunn jaw-winking synkinesis (MGJWS) is characterized by eyelid ptosis, which disappears during jaw movement. Familial MGJWS is an extremely rare condition. Some authors suggested that MGJWS is due to neural misdirection in the brainstem whereas others suggested that aberrant reinnervation or ephapse may be responsible for synkinetic activity. Pathogenesis of this condition is therefore still unclear.

**Methodology/Principal Findings:**

To investigate pathogenetic mechanism in familial MGJWS we performed neurophysiological (EMG, Blink Reflex, Recovery cycle of the R2 component of the blink reflex, Masseter inhibitory reflex, BAEPS and kinematic analysis) and neuroradiological (MRI, Diffusion Tensor Imaging) investigations in a member of a multigenerational family with autosomal dominant Marcus Gunn jaw-winking synkinesis (MGJWS). Kinematic analysis of eyelid and jaw movements disclosed a similar onset and offset of the eyelid and jaw in both the opening and closing phases. The excitability of brainstem circuits, as assessed by the blink reflex recovery cycle and recovery index, was normal. Diffusion Tensor Imaging revealed reduced fractional anisotropy within the midbrain tegmentum.

**Conclusions/Significance:**

Kinematic and MRI findings point to a brainstem structural abnormality in our familial MGJWS patient thus supporting the hypothesis of a neural misdirection of trigeminal motor axons to the elevator palpebralis muscle.

## Introduction

Marcus Gunn jaw-winking synkinesis (MGJWS) is characterized by eyelid ptosis, which disappears during jaw movement. MGJWS is usually considered a sporadic condition, though familial cases have been documented [1]. Mutations in the TUBB3 gene, encoding beta-tubulin isotype III, have been identified in a spectrum of disorders characterised by central nervous system and oculomotor abnormalities, including MGJWS [2].

Neurophysiological studies have suggested that the Marcus-Gunn phenomenon may be due to a congenital neural misdirection of trigeminal motor axons of the external pterygoid muscle (responsible for jaw depression and protraction) to the elevator palpebralis muscle [3]. Other authors have instead suggested that the Marcus-Gunn phenomenon may originate from aberrant reinnervation at the brainstem level rather than at the peripheral level [4]. Aberrant reinnervation may be secondary to brain damage occurring during embryonic development or may be caused by ephaptic transmission between adjacent axons.

Familial MGJWS is extremely rare. More information on the clinical, neurophysiological and neuroimaging features of patients with familial MGJWS is needed to provide an insight into the pathogenesis of this condition.

In the present paper we describe the clinical, neurophysiological (kinematic analysis of the jaw-winking synkinesis and Recovery cycle of the R2 component of the blink reflex) and neuroimaging (Magnetic Resonance Imaging - MRI, Diffusion Tensor Imaging -DTI) findings in a member of a multigenerational family with autosomal dominant MGJWS.

## Subjects and Methods

### Subjects

A 37-year-old man presented with right eyelid ptosis, which disappeared when the patient opened his mouth and reappeared when he closed his mouth. Synkinetic activation of the left orbicularis oculi muscle when attempting to voluntarily open the right eyelid was also present. Symptoms had been present since birth. The subject’s family history revealed that both his father and two paternal uncles were affected by the same disorder.

The neurological examination disclosed no further abnormality while the ophthalmological examination revealed eterophory at distance and vertical strabismus. Electromyography (EMG) recordings from the facial muscles were normal, as were the brainstem auditory evoked potentials.

The control group for the neuroradiological examinations comprised 29 age- and sex-matched healthy subjects (41±5.6 years). Twenty out of the 29 healthy volunteers were also involved in the neurophysiological investigations (age: 37±5.0 years). Both the MGJWS patient and the healthy subjects gave their written informed consent to the experimental procedures, which were approved by the Ethics Committee of University of Rome “Sapienza” and conducted in accordance with the Declaration of Helsinki.

### Neurophysiological Assessment

The kinematics of the eyelid and jaw movement was recorded using a 3D optoelectronic motion system (SMART motion system, BTS, Milan, Italy). The displacement of the reflective marker taped on the centre of the lower margin of the upper eyelid and on the mental prominence of the jaw in three-dimensional space was reconstructed off-line by a dedicated software (SMART Analyzer, BTS, Milan, Italy). Onset, offset, duration, peak velocity and amplitude were automatically computed for each downward and upward eyelid and jaw movement [5], [6]. Differences between the onset of eyelid and jaw movement were calculated. The Paired-sample T test was used to compare the onset of the upper eyelid and jaw movements during jaw opening and closing. Pearson’s correlation coefficient was used to analyse movement duration of eyelid and jaw opening and closing movements.

The blink reflex, the masseter inhibitory reflex and the blink reflex recovery cycle were performed according to previously described techniques [7], [8]. The blink reflex recovery cycle was studied by delivering paired electrical shocks to the supraorbital nerve at interstimulus intervals (ISI) of 250 and 500 ms. The R2 recovery index was also calculated.

### Neuroimaging Assessment

All the subjects underwent an MR imaging examination on a 3T scanner system (Intera Achieva, Philips Medical Systems, Best, The Netherlands). Diffusion-weighted images were corrected for head motion and eddy current distortions using FDT (FMRIB’s Diffusion Toolbox 2.0) [9], after which brain tissue was segmented using BET. Tensor fitting employed a constrained non-linear least squares procedure (CAMINO) [10], followed by Fractional Anisotropy (FA) map estimation. Voxelwise statistical analysis of the data was carried out using Tract-Based Spatial Statistics (TBSS) [11]. TBSS projects all the subjects’ FA data onto a mean FA tract skeleton, before applying voxelwise cross-subject statistics. The latter included full correction for multiple comparisons over space using permutation-based non-parametric inference (50,000 permutations). P-values were calculated and corrected for multiple comparisons using the “2D” parameter settings with threshold-free cluster enhancement [12].

## Results

**Figure 1:**
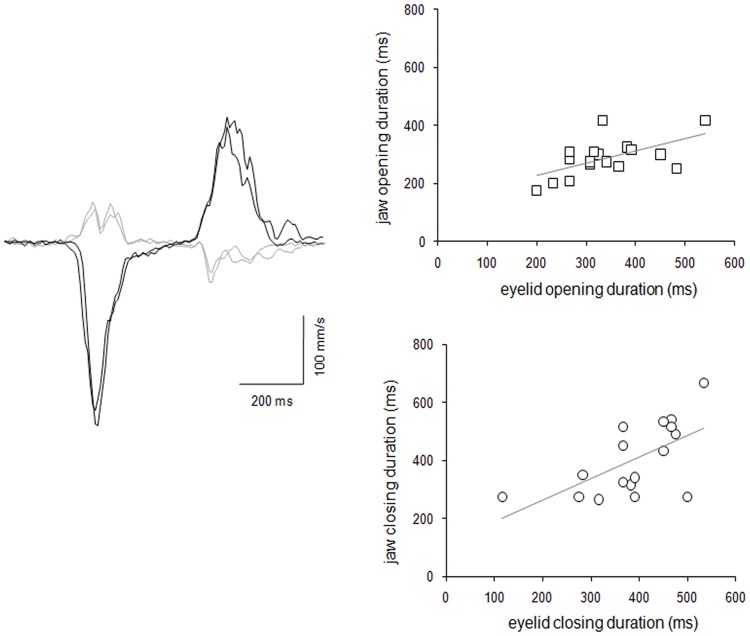
Left: velocity curves for eyelid-jaw synkinesis in a patient with familial Marcus Gunn jaw-winking synkinesis (MGJWS). Black line represents velocity trace of jaw movement, gray line represents velocity trace of eyelid movement. The x-axis corresponds to time (ms) and the y-axis to velocity (mm/s). The first peak in the velocity trace corresponds to the peak velocity of the opening phase, the second peak-to-peak velocity of the closing phase. Right: Upper panel. Correlation between jaw opening duration and eyelid opening duration expressed in milliseconds. Lower panel. Correlation between jaw closing duration and eyelid closing duration expressed in milliseconds.

The patient’s clinical examination showed that jaw opening and closing movements elicited eyelid-jaw synkinetic activity. The kinematic analysis did not reveal any significant differences between the onset of eyelid and jaw opening and the offset of eyelid and jaw closing (p = 0.34). Pearson’s correlation coefficient disclosed a significant correlation between movement duration of the eyelid and jaw during both the opening (r = 0.58, r^2^ = 0.33, p = 0.01) and closing phases (r = 0.61, r^2^ = 0.37, p = 0.009) (figure 1). The patient’s blink reflex test and the masseter inhibitory reflex yielded normal findings (R1 mean latency = 10.2 ms, ipsilateral R2 mean latency = 36.0 ms and contralateral R2 mean latency = 36.3 ms; ipsilateral SP1 mean latency 11.7 ms and SP2 mean latency = 38.5 ms, contralateral SP1 mean latency 12.0 ms and SP2 mean latency = 39.1 ms). The blink reflex recovery cycle and R2 recovery index was comparable to those of a group of 20 healthy subjects (R2 area expressed as percentage of the unconditioned R2 = 18% at 250 ms, 35% at 500 ms; R2 recovery index as a percentage = 26.5).

**Figure 2:**
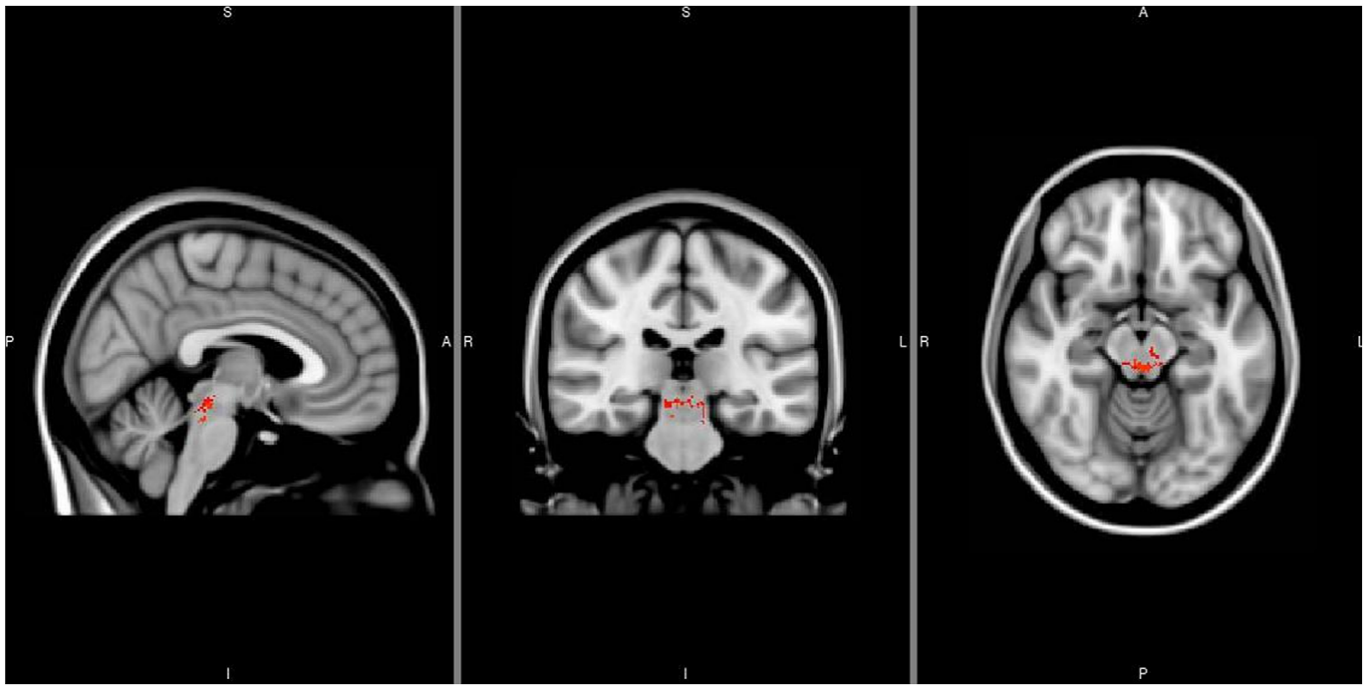
Significant reduction in FA (voxels in red) in the brainstem of a patient with familial Marcus Gunn jaw-winking synkinesis (MGJWS) overlaid on the MNI T1 non-linear template image. The FA alterations are localized within the midbrain tegmentum, the reticular formation and the central tegmental tracts.

Conventional T1, T2 and FLAIR images were normal in all the brain regions. TBSS revealed a reduction in FA in the oculomotor nuclear complex, reticular formation and central tegmental tract areas (figure 2). No MD abnormalities were detected.

## Discussion

The kinematic analysis performed in this patient showed that the MGJWS was characterized by a similar onset and offset of the eyelid and jaw movements in both the opening and closing phases. We also found normal brainstem excitability, as assessed by the blink reflex recovery cycle and recovery index. Lastly, the neuroimaging investigation disclosed distinctive abnormalities in the FA value of the brainstem.

In normal subjects, eyelid activation involves three neural pathways: the elevator palpebralis muscle, which is innervated by the oculomotor nerve, raises the upper eyelid; gentle upper eyelid closure depends on the inhibition of motor axons directed to the elevator palpebralis muscle, which occurs in the brainstem at the level of trigeminal motor nucleus; forced eyelid closure depends on the orbicularis oculi muscle, which is innervated by the facial nerve. Previous observations of MGJWS have suggested that jaw and eyelid synkinetic activity is due to ephaptic activation of motorneurons directed to the elevator palpebralis muscle when the pterigoideus muscle is activated [13]. Ephaptic transmission propagates neural impulses between adjacent cells through electrotonic mechanisms, thereby resulting in crosstalk between adjacent axons [13], and ephaptic activity between mammalian axons occurs as a transient phenomenon following acute injury of normal peripheral nerves [14]. Spontaneous activity in ephaptically interacting pairs of fibres generally persists for a short period of time, with ephaptic activity in the excited fibres being characterized by lack of uniformity in the propagation of impulses away from the ephapse in two directions [14]. In the patient we studied, the kinematic observation over time of stereotyped, reproducible and stable eye-jaw synkinetic movements, in both the opening and closing phases, indicates that an ephaptic mechanism is unlikely to be involved in the pathophysiology of familial MGJWS. Our observations therefore suggest that MGJWS is due to neural misdirection of motor axons in the brainstem. A limitation of the study is that as in the patient we studied we did not perform polygraphic needle EMG recordings from the elevator palpebralis muscle [8] and pterygoideous muscle, we cannot completely ruled out the possibility that ephaptic transmission could be responsible of the synkinetic activation.

Reduced FA in the brainstem of MGJWS, however, further supports the hypothesis of neural misdirection in the brainstem. The FA alterations were localized within the midbrain tegmentum, where the oculomotor nuclear complex, the reticular formation and the central tegmental tracts are located. These observations point to a structural basis of MGJWS. FA is highly sensitive to subtle differences in white matter architecture at the microstructural level and FA alterations reflect changes in the alignment of cellular structures within fiber tracts and in their structural integrity [15]–[17]. Moreover, the sensitivity of DTI-based techniques has been confirmed in congenital brain anomalies [18]. Our MGJWS patient also had synkinetic activation of the left orbicularis oculi muscle when attempting to voluntarily open the right eyelid. The bilateral neuroradiological FA abnormality we found in the patient is consistent with the right-to-left elevator palpebralis-orbicularis oculi muscles synkinetic activity. Although there is some evidence of synkinetic activation of the orbicularis oculi muscle during jaw movements, possibly due to peripheral anastomosis (trigemino-facial associated movement) [19], to our knowledge, this is the first report showing a patient with isolated MGJWS affected by involuntary contraction of the orbicularis oculi muscle due to the activation of the contralateral elevator palpebralis muscle. Since the FA alterations we observed may also be due to variance inflation or to a heavier tailed distribution, we cannot exclude the possibility that this patient was a false-positive case, though the congruence between the localization of the altered FA values and the clinico-neurophysiological findings suggests this hypothesis is unfounded.

The blink reflex recovery cycle is a technique specifically designed to evaluate the excitability of the orbicularis oculi reflex in the brainstem [7]. Previous studies [20], [21] have reported that the blink reflex recovery cycle is abnormal in patients with dystonia affecting sites other than the facial muscles, a finding ascribed to an abnormal control of the interneuronal networks mediating the blink reflex. They also concluded that the close proximity to the cranial muscles played an important role in determining the extent of abnormal interneuron function. However, although neural circuits underlying blink reflex and the eyelid-jaw synkinesis do not overlap, the finding of a normal blink reflex recovery cycle suggests that eyelid-jaw synkinetic activity and right-to-left synkinesis unlikely depend on widespread brainstem hyper-excitability.

To our knowledge, this is the first study conducted by means of both neurophysiological and neuroradiological techniques demonstrating a structural abnormality in the brainstem, thus supporting the hypothesis of a neural misdirection of trigeminal motor axons to the elevator palpebralis muscle.
